# Non-targeted Metabolomics Analysis Based on LC–MS to Assess the Effects of Different Cold Exposure Times on Piglets

**DOI:** 10.3389/fphys.2022.853995

**Published:** 2022-04-05

**Authors:** Yong Chen, Hong Ji, Jingru Guo, Yan Chen, Wenjie Li, Shengping Wang, Li Zhen

**Affiliations:** ^1^ College of Animal Science and Veterinary Medicine, Heilongjiang Bayi Agricultural University, Daqing, China; ^2^ Hunan Institute of Microbiology, Changsha, China

**Keywords:** piglets, non-targeted metabolomics, cold exposure, LC–MS, liver

## Abstract

Pigs are susceptible to low temperature conditions, and cold stress causes metabolic changes in the body to increase heat production as an adaption to adverse environments. To characterize and validate different metabolites in piglet livers at different cold exposure times, sixteen 30-day-old male weaned piglets with similar weights were randomly divided into four groups: the normal temperature group (24 ± 2°C, NT) and cold exposure (4 ± 2°C) 2-h group (CS2), 6-h group (CS6), and 12-h group (CS12). At the end of the experiment, the liver samples were analyzed using systemic non-targeted metabolomics. Eight known differentially abundant metabolites (farnesyl pyrophosphate, isocitrate, triethanolamine, phenylethylamine, deoxynosine, citric acid, maltotriose, and epinephrine) were observed between the CS groups and the control group in positive and negative ion modes. The eight main differentially abundant metabolites involved in seven metabolite classifications. Metabolic pathways and enrichment analyses revealed that the pathways involved three KEGG pathway classifications. Most of the pathways were related to amino acid or energy metabolism. Moreover, the metabolic pathways were not identical under different cold exposure times, with those following 2 and 6 h of cold exposure more related to carbohydrates and energy production and those following 12 h of cold exposure more related to the metabolism connected with epinephrine. Thus, under different cold exposure times, the metabolite profiles and metabolic pathways differed.

## Introduction

Low and high ambient temperature conditions are very common in livestock production and induce different levels of stress (Young, 1981). Pigs are sensitive to temperature changes, especially to low temperature, and cold stress is an important contributing factor to piglet morbidity and mortality (Muns et al., 2016).

Under low ambient temperature, the energy requirements increased dramatically (Chmielowiec-Korzeniowska et al., 2012). Non-shivering thermogenesis is important in cold-stressed pigs due to a lack of brown adipose tissue (White et al., 2015; Zhang et al., 2017). Herpin et al. (2005) noted that the muscles of pigs have high oxidative potential and oxygen consumption to increase heat production for body thermal regulation. Cold stress causes metabolic changes in the body and decreases the gain rate and feed efficiency of pigs as metabolic responses to cold conditions for heat production (Birkelo et al., 1991). Depending on the intensity of the exposure, cold stress can trigger different stress responses in cells. Sonna et al. (2002) indicated that more than 17 genes (apoptosis specific protein, IL-8, HSP25, ATPase, etc.) upregulated or downregulated under cold stress, and most were downregulated to adapt to the metabolic demands of the body. A study on puffer fish under cold conditions observed the upregulation of bile salt export pumps involved in the transport of oligosaccharides (D-maltose and maltotriose) which are beneficial for energy requirement (Wen et al., 2019). The muscle gene expression profile of pigs suffering cold stress showed that the differently expressed genes were mainly distributed in the cytoplasm, mitochondrion, organelle envelope, and ribosomal subunit and enriched pathways primarily in ribosomes, fatty acid metabolism, RNA transport, and metabolic pathways (Zhang et al., 2017). Under cold conditions, the activity of the sympathetic system and hormones regulates the physiology and metabolism of pigs (Young, 1981). Gebregeziabhear et al. (2015) reported that pigs in a negative condition had higher cortisol concentrations and lower growth rates. Nie et al. (2021) pointed out that stress hormones (epinephrine, norepinephrine, and glucocorticoids) are important for the metabolism of brown adipose tissue to maintain thermogenesis. The secretions of adrenal corticosteroids and some hormones stimulated by cold stress also affect the body immune function based on the differentiation, proliferation, or gene expression of immune cells (Zhang et al., 2017). Moreover, cold conditions change the hair coat, surface tissues, appetite, and digestive function (Young, 1981). Many cellular processes are regulated at the level of metabolites, such as cell signaling, energy transfer, and intercellular communication. Metabolites reflect the environment of cells and are closely related to the nutritional status of cells, the role of drugs and environmental pollutants, and other external factors.

Metabonomics is an important technique for systems biology and involved the measurement of metabolites that are the end products of biological processes. High-throughput detection and data processing have detected the low molecular weight metabolites of a certain organism or cell in a specific physiological period, and some of these data have been indexed in databases. A metabonomic study on pig cecal content revealed that short-term cold stress changed the concentration of certain metabolite concentrations, and among the identified metabolites, creatinine and oxypurinol mainly occur in the blood and are related to physiological activities such as muscle contraction (Yang et al., 2021). In a study of rats suffering from cold stress, metabolomic profiling was performed, and important biochemical responses (e.g., tricarboxylic acid cycle, gut microbiota) to acute and chronic cold stress were identified (Wang et al., 2009).

The main objectives of this study were to characterize the metabolic profiles of the piglet liver under different cold exposure times and determine the effects of cold exposure times on the metabolome and metabolic pathways. The identification of different metabolites and pathways, especially at different times, is helpful for better understanding the adaptation process under different cold exposure conditions, and such data can be used for improving climate adaptability in pig production.

## Materials and Methods

### Animals, Experimental Design, and Sampling

Sixteen 30-day-old male weaned piglets with similar weights (22.9 ± 0.2 kg) were randomly divided into four groups: a normal temperature group (24 ± 2°C, NT) and a cold exposure (4 ± 2°C) 2-h group (CS2), 6-h group (CS6), and 12-h group (CS12). The piglets in all groups were reared in artificial intelligence climate chambers for 7 days with a 12/12-h light/dark cycle (light from 8:00 a.m. to 8:00 p.m.). The temperature was maintained at 24 ± 2°C, and the relative humidity was 40–50%. Then, the piglets in the cold exposure groups were exposed to 4 ± 2°C conditions for 2, 6, or 12 h in artificial intelligence climate chambers, while the normal temperature control group was kept at 24°C. All piglets were fed the same commercial pellet feed and water, which were provided *ad libitum*. At the end of the experiment, all piglets were anesthetized and immediately euthanized. Liver samples (3–5 g) were then frozen with liquid nitrogen and stored in a -80 °C freezer until analysis by LC–MS metabonomics.

### Extraction of Metabolites

Liver samples (50 mg) were added to 1,000 μl of the extraction solution (methanol: acetonitrile: water = 2: 2: 1 (V/V), including 2 μg/ml of the internal standard L-2-chlorophenylalanine), vortexed, homogenized, and centrifuged at 13,800 ×g for 10 min at 4°C. Then, 400 μl of the supernatant was placed in an EP tube for vacuum drying, 200 μL of 50% acetonitrile was added for further dissolution, and then, the samples were vortexed and centrifuged at 16,200 ×g for 15 min at 4°C. Then, 75 μL of the supernatant was added to the sampler bottle (Dunn et al., 2011).

### LC-MS/MS Analysis Procedure and Conditions

The supernatant in the sampler bottle was analyzed using a 1,290 Infinity series UHPLC System equipped with an UPLC BEH Amide column (2.1 × 100 mm, 1.7 μm, Waters) (Agilent Technologies) with a mobile phase comprising 25 mmol L^−1^ ammonium acetate and 25 mmol L^−1^ ammonia hydroxide in water (pH = 9.75) (A) and acetonitrile (B). The analysis was carried out with an elution gradient as follows: 0 ∼ 0.5 min, 95% B; 0.5 ∼ 7.0 min, 95 ∼ 65% B; 7.0 ∼ 8.0 min, 65 ∼ 40% B; 8.0 ∼ 9.0 min, 40% B; 9.0 ∼ 1 min, 40 ∼ 95% B; and 9.1 ∼ 12.0 min, 95% B. The column temperature was 25°C, and the injection volume was 2 μL (positive) or 2 μL (negative).

A triple TOF 6600 mass spectrometer (AB Sciex) was used to acquire MS/MS spectra on an information-dependent basis (IDA). The acquisition software (Analyst TF1.7, AB Sciex) continuously evaluates the full-scan survey MS data as it collects and triggers the acquisition of MS/MS spectra depending on preselected criteria. The ESI source conditions were as follows: Gas 1 was 60 psi, Gas 2 was 60 psi, curtain gas was 35 psi, source temperature was 600°C, declustering potential was 60 V, and ion spray voltage floating (ISVF) was 5000 V or -4000 V in positive or negative modes, respectively.

### Data Analysis and Annotation

MS raw data files were converted to the mzXML format by ProteoWizard and processed by the R package XCMS (version 3.2). The process includes peak deconvolution, alignment, and integration. MinFrac and cut off were set as 0.5 and 0.3, respectively. The in-house MS2 database (based on HMDB, PubChem, KEGG, and METLIN) was applied for metabolite identification.

## Results and Discussion

Non-targeted metabolomics data from the piglet liver were collected by a UHPLC-QTOF-MS analysis in positive and negative modes. In total, 993 metabolite ion features were detected in the liver samples in positive and negative ion modes.

### Principal Component Analysis

**FIGURE 1:**
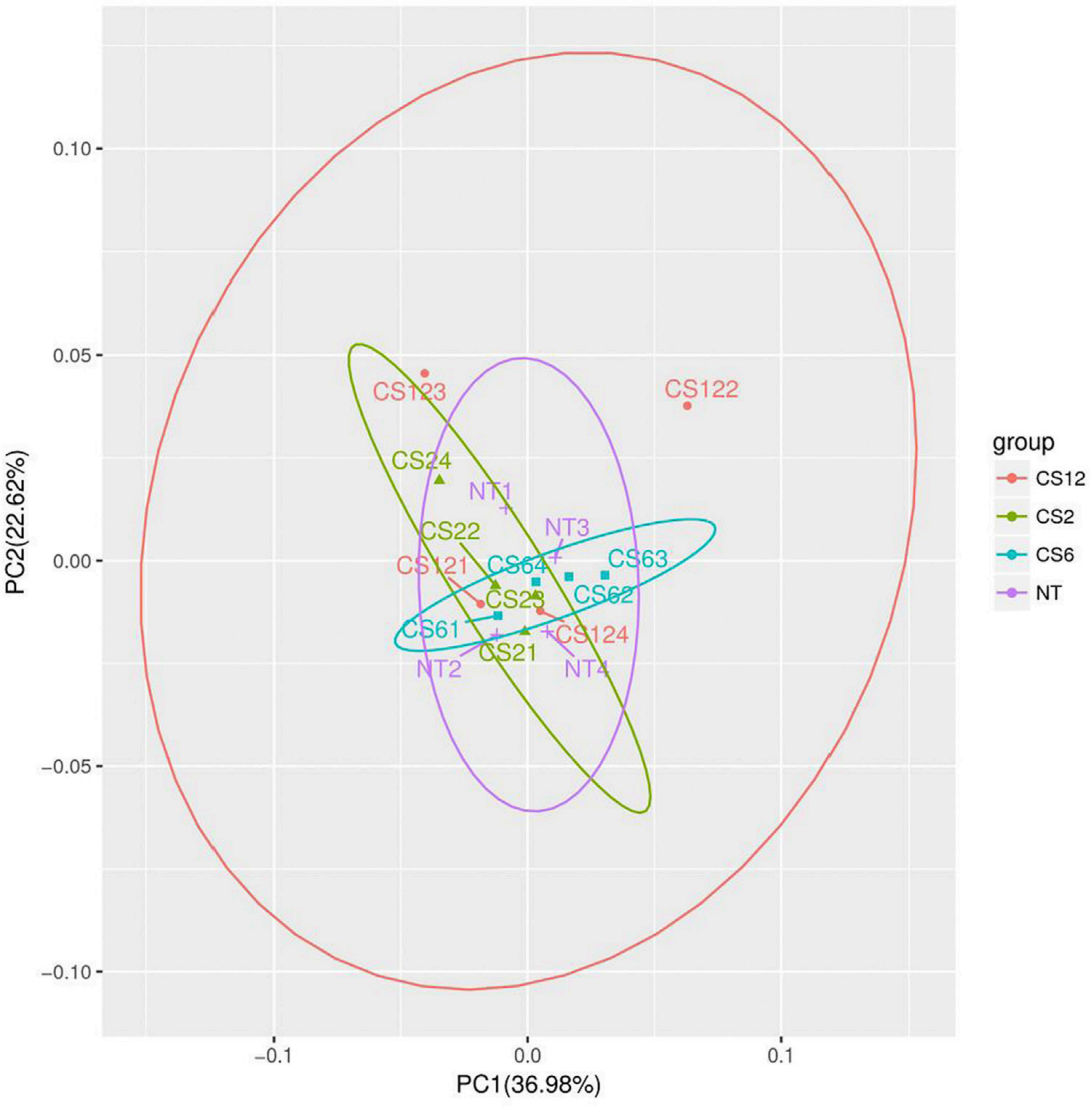
PCA score plots of different cold exposure duration groups. Note: NT=normal temperature group (24±2°C), CS2=cold exposure (4±2°C) 2-hour group, CS6=cold exposure 6-hour group, and CS12=cold exposure 12-hour group; NT1 ∼ 4 in the figure corresponded to the four samples of the NT group (24±2°C), similar to the CS2, CS6, and CS12 groups.

To obtain an overview of the chemical changes in the piglet liver during different cold exposure durations, the data were analyzed by unsupervised PCA. The PCA scores are shown in Figure 1. The data showed one principal component (PC) (PC1), two PCs (PC1 and PC2), and three PCs (PC1, PC2, and PC3), which accounted for 37.0, 59.2,, and 70.3% of the variation, respectively.

### Orthogonal Partial Least Squares Discriminant Analysis

**FIGURE 2:**
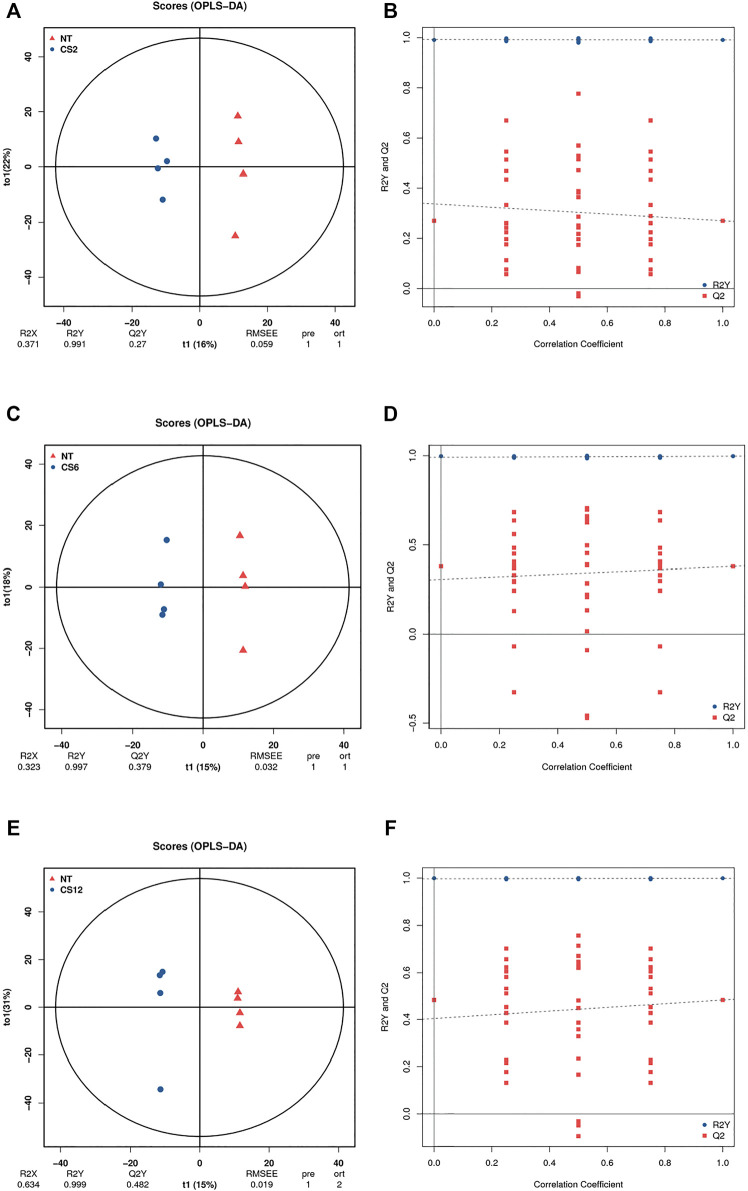
OPLS-DA score plot diagrams **(A)**, NT *vs* CS2; **(C)**, NT *vs* CS6; **(E)**, NT *vs* CS12 and permutation test results **(B)**, NT *vs* CS2; **(D)**, NT *vs* CS6; **(F)**, NT *vs* CS12.

To further verify the significant difference between the control and cold exposure groups, we used the supervised OPLS-DA multivariate method for each of the two comparisons. The OPLS-DA score plots indicate the good fitness and high predictability of the model, with high statistical values of R^2^Y and Q^2^. The parameters of R^2^Y and Q^2^Y for CS2-NT, CS6-NT, and CS12-NT were 0.991 and 0.27, 0.997 and 0.379, and 0.999 and 0.482, respectively (Figures 2A,C,E). The permutation test results showed that all Q^2^ points from left to right were lower than the original red Q^2^ points on the right side, indicating that the model was robust and reliable without overfitting (Figures 2B,D,F). The control and cold exposure groups could be clearly differentiated, suggesting that the metabolic profiles changed significantly and could be exploited in the subsequent analysis.

### Identification and Analysis of Differentially Abundant Metabolites

**FIGURE 3:**
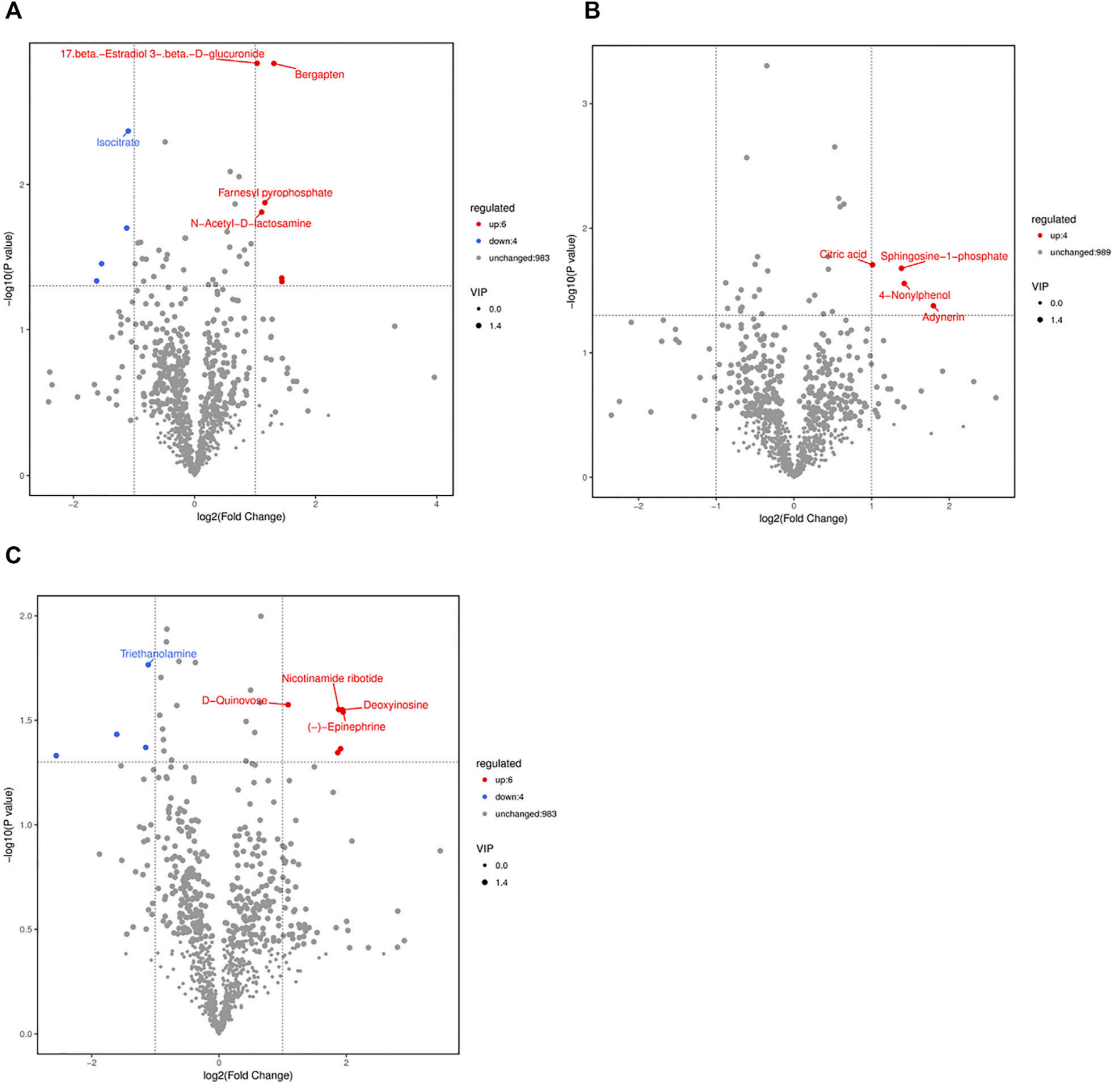
Volcano plots of the NT and CS groups based on spectral data from the ion modes **(A)**, NT *vs* CS2; **(B)**, NT *vs* CS6; **(C)**, NT *vs* CS12. Note: one point in the graph corresponds to a metabolite, and the size of the points represents the VIP value of the OPLS-DA model. Red indicates upregulated metabolites, blue indicates downregulated metabolites, black indicates metabolites that were not significantly different between the CS and NT groups.

According to the supervised manipulations, potential variables were chosen as differentially abundant metabolites based on VIP >1, fold change >2, and *p* < 0.05 ((Thévenot et al., 2015)). There were 10 differentially abundant metabolites (6 upregulated and 4 downregulated) in the CS2 group, 4 upregulated differentially abundant metabolites in the CS6 group, and 10 differentially abundant metabolites (6 upregulated and 4 downregulated) in the CS12 group compared with the control group (NT) (Table 1 and Figure 3).

**TABLE 1:** Differential metabolites observed in the samples.

RT	m/z	MS2 name	Fold change	*p* Value	VIP	KEGG annotation	KEGG pathway
NT *vs*. CS2							
31.153	281.14	Phenylethylamine	0.33	0.05	1.95	C05332	ko00360
42.652	251.04	Isocitrate	0.47	0.00	2.27	C00311	ko00020, ko01230, ko04922, ko05230, ko01210, ko00630, ko01100, ko01200
164.307	150.11	Triethanolamine	0.46	0.02	2.03	C06771	ko00564
171.894	438.27	Sphingosine-1-phosphate	2.71	0.04	1.85	—	—
277.881	507.22	17beta-Estradiol 3-beta-D-glucuronide	2.05	0.00	2.36	—	—
285.969	351.17	Fluoxetine	2.72	0.05	2.04	—	—
287.492	310.06	Lys-Ser	0.34	0.04	2.00	—	—
287.838	280.05	Bergapten	2.48	0.00	2.43	C01557	—
293.947	399.16	N-Acetyl-D-lactosamine	2.15	0.02	2.07	—	—
320.527	419.07	Farnesyl pyrophosphate	2.24	0.01	2.05	C00448	ko01100, ko00900, ko00981, ko00100
NT *vs*. CS6							
25.273	219.18	4-Nonylphenol	2.68	0.03	2.09	—	—
42.253	515.30	Adynerin	3.47	0.04	1.99	—	—
171.894	438.27	Sphingosine-1-phosphate	2.61	0.02	2.09	—	—
271.357	173.01	Citric acid	2.02	0.02	2.06	C00158	ko00630, ko04742, ko00020, ko01100, ko00250, ko01200, ko01210, ko01230, ko04922, ko05230
NT *vs*. CS12							
37.468	335.22	Amitraz	0.33	0.04	2.07	—	—
39.258	384.20	Epinephrine	3.86	0.03	2.22	C00788	ko04080, ko04923, ko01100, ko04261, ko00350, ko04024, ko04924
40.765	263.02	3-Methoxy-4-hydroxyphenylglycol sulfate	3.64	0.05	2.03	—	—
40.891	321.17	Phenoxybenzamine	0.45	0.04	1.96	—	—
164.307	150.11	Triethanolamine	0.46	0.02	2.11	C06771	ko00564
171.648	339.06	Met-Leu	3.76	0.04	2.04	—	—
190.562	163.06	D-Quinovose	2.12	0.03	2.19	—	—
353.861	333.06	Nicotinamide ribotide	3.68	0.03	2.07	—	—
469.66	522.20	Maltotriose	0.17	0.05	2.07	C01835	ko02010, ko04973

Note: NT, normal temperature group (24 ± 2°C); CS2, cold exposure (4 ± 2°C) 2-h group; CS6, cold exposure 6-h group; and CS12, cold exposure 12-h group.

Among the NT and cold exposure groups, there were 8 known differentially abundant metabolites, which were primarily important intermediate metabolites for body metabolism.

#### Isocitrate

Isocitrate is important as an electron donor for NADP^+^-dependent isocitrate dehydrogenase to reduce NADP^+^ to NADPH (Minich et al., 2003). In mitochondria, NADPH is critical for antioxidant defense to reduce the byproducts of respiration (Lin et al., 2011). Cold stress may elevate the metabolic rate and induce high reactive oxygen species levels (Şahin and Gümüşlü, 2004). In our study, the isocitrate level was downregulated in the 2-h cold exposure group compared with the NT group; however, its level did not show a change in the 6- and 12-h cold exposure groups compared with the NT group. The different isocitrate levels showed that 2 h of cold exposure may result in oxidative damage to the cells, although the injured state could be relieved with additional time for metabolic adjustments of the body. A cold stress study on rats showed similar changes, with decreases observed in the urinary excretion levels of isocitrate in Sprague–Dawley rats exposed to -10 °C for 2 h (Wang et al., 2007). However, an alternative 4°C treatment and a normal temperature treatment of Wistar rats for 2 weeks did not result in changes in plasma isocitrate (Yang et al., 2015). The different effects are probably due to the different cold stress times or conditions.

#### Farnesyl Pyrophosphate

FPP is endogenously synthesized and represents a key intermediate for the biosynthesis of steroids, carotenoids, and polyisoprenoids. G protein-coupled receptors (GPRs) are cell surface receptors that play varied roles in pathophysiological processes by transmitting cell signals (Oh et al., 2008). In GPR92-expressing cells, FPP directly interacts with GPR92 and induces immediate responses, such as Ca^2+^ mobilization, inositol phosphate and cAMP accumulation, and phospho-ERK elevation (Oh et al., 2008). In our study, FPP was upregulated in the 2-h cold exposure group compared with the NT group, which was probably related to the ability of FPP to accelerate body metabolism at the first stage of cold stress. Moreover, FPP is positively related to the biosynthesis of some non-steroidal isoprenes that are important for intracellular antioxidants (Qi et al., 2010).

#### Phenylethylamine

PEA has weak sympathomimetic traits, stimulates motor activity, and is a potentially common mediator of amphetamine and stress (Durden et al., 1973). In humans and rats, stress increases the impaired excretion of PEA (Snoddy et al., 1985). At the beginning of cold stress, it was speculated that depression led to the impaired excretion of PEA, which caused the downregulation of PEA in the 2-h cold exposure group compared with the NT group. Along with the improvement of depression, the pigs showed tolerance toward cold stress. Therefore, there was no difference in PEA between the 6- or 12-h cold exposure groups and the NT group.

#### Citric Acid

Citric acid is a component of the Krebs cycle, and it is affected by many factors, such as stressors and hormone and dietary protein levels (Hill et al., 1961). Hill et al. (1961) noted that the citric acid levels in the blood increased when chickens were subjected to heat stress, cold stress, starvation stress, or ACTH administration; however, the citric acid concentration did not show these changes if the dietary protein level was lower than 10%, which probably reflects that a protein or some amino acids are required for synthetases related to citric acid (Hill and Baker, 1961). Studies on rabbits showed that administering citrate was beneficial to improving the antioxidant function of the liver and reduced the effects of cold and heat stress, although changes in acupuncture stress were not observed (Liao et al., 1980). In the present study, the level of citric acid was upregulated only in the 6-h cold exposure group compared with the NT group, which may be associated with the effects of different cold conditions. The pigs suffered longer cold stress times (24, 48 h, and 5 days), and variations in the citric acid level were not detected (Zhang et al., 2017; Zhang et al., 2020). Marai and Habeeb (2010) noted that the citric acid contents also decreased in the milk of Friesian cows and semen of buffalos under high-temperature conditions.

#### Maltotriose

Maltotriose is a product of starch digestion and can enhance energy metabolism. Maltose and maltotriose are the first carbohydrates accumulated under cold conditions, followed by the others (hexose phosphates, fructose-6-phosphate, glucose-6-phosphate, galactose-6-phosphate, and mannose-6-phosphate) (Purdy et al., 2011). A cold stress response study on juvenile pufferfish showed that the liver maltotriose level was upregulated at 12°C for 24 h compared with that in the fish at 26°C (Wen et al., 2019). However, juvenile yellow drums did not show the same change under cold stress, and the liver maltotriose level of fish in 8°C water for 2 weeks was not upregulated or downregulated compared with that of fish in 16°C water (Jiao et al., 2020). Moreover, the liver maltotriose level of starved yellow drums was downregulated compared with that of yellow drums fed under normal temperature conditions (Jiao et al., 2020). In our study, the liver maltotriose of the pigs did not show differences between the 2- or 6-h groups and the NT group; however, the liver maltotriose level of pigs in the 12-h group was downregulated. The reason for these findings may be the non-acute cold condition. However, the body starch level decreased with the cold exposure duration, which caused a decrease in maltotriose, and this change may affect energy metabolism.

#### Epinephrine

Epinephrine is secreted by the adrenal gland, and it is necessary for appropriate responses to various stressors and is beneficial for metabolic regulation in the body. Heat stress increases the body maintenance requirements in rodents, poultry, sheep, and cattle and elevates the level of epinephrine (Baumgard and Rhoads, 2013). Pigs at 18 weeks in the heat stress group (37°C for 9 h and 23°C for 15 h) had higher epinephrine concentrations than those in the control group (23°C for 24 h) (Lan and Kim, 2018). However, the plasma epinephrine level of large white pigs (6 h of age) at 25°C (cold) was not significantly different from that of newborn pigs at 34°C (thermoneutral) for 48 h (Berthon et al., 1996). The plasma epinephrine level of large white pigs aged 6–10 weeks was not affected by cold stress. The change in epinephrine levels is likely linked to severe stress (Barrand et al., 1981; Young, 1981). Ambient temperatures of less than 20°C have a significant effect on the sympathetic adrenal system of humans. Under severe stress (<15°C), functional alterations were observed in the adrenal medulla (epinephrine). Acute cold stress increased plasma epinephrine concentrations of shorn sheep by two- and five-fold and increased the urinary excretion of epinephrine (Young, 1981). In our study, the liver epinephrine level did not show a difference between the 2/6-h group and the NT group; however, the epinephrine level in the 12-h group was upregulated. These findings may not have been caused by the acute cold conditions but rather by the decreased body energy metabolism under increased cold exposure time.

Except for the differential metabolites between the cold exposure groups and the control, many of the identified metabolites did not show differences; however, some differential metabolites have been observed under different cold stress conditions in previous studies. For example, deoxyuridine, indoleacetic acid, histidine, phenylacetylglycine, enterostatin, methoxyacetic acid, dihydroxyfumarate, creatinine, and oxypurinol were identified as differential metabolites in the serum or cecal mucosa samples under long-term cold stress conditions (Zhang et al., 2020; Yang et al., 2021). Researchers have pointed out that metabolites are mainly associated with the physiological activities of energy metabolism and muscle contraction (Zhang et al., 2020; Yang et al., 2021).

### KEGG Annotation of Differentially Abundant Metabolites

**FIGURE 4:**
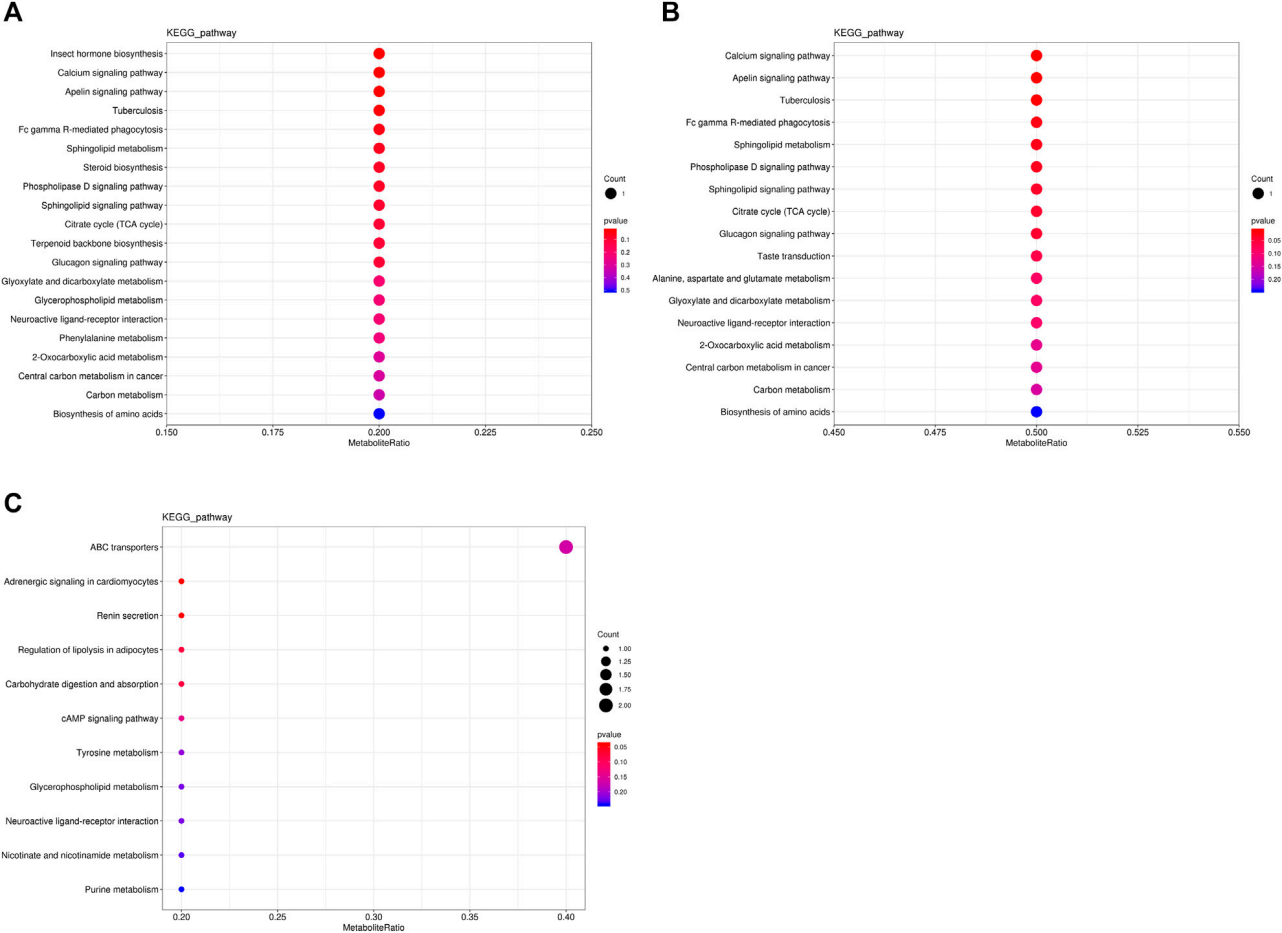
KEGG pathway-enriched dot plots of differentially abundant metabolites. **(A)**, NT *vs* CS2; **(B)**, NT *vs* CS6; and **(C)**, NT *vs* CS12.

**FIGURE 5:**
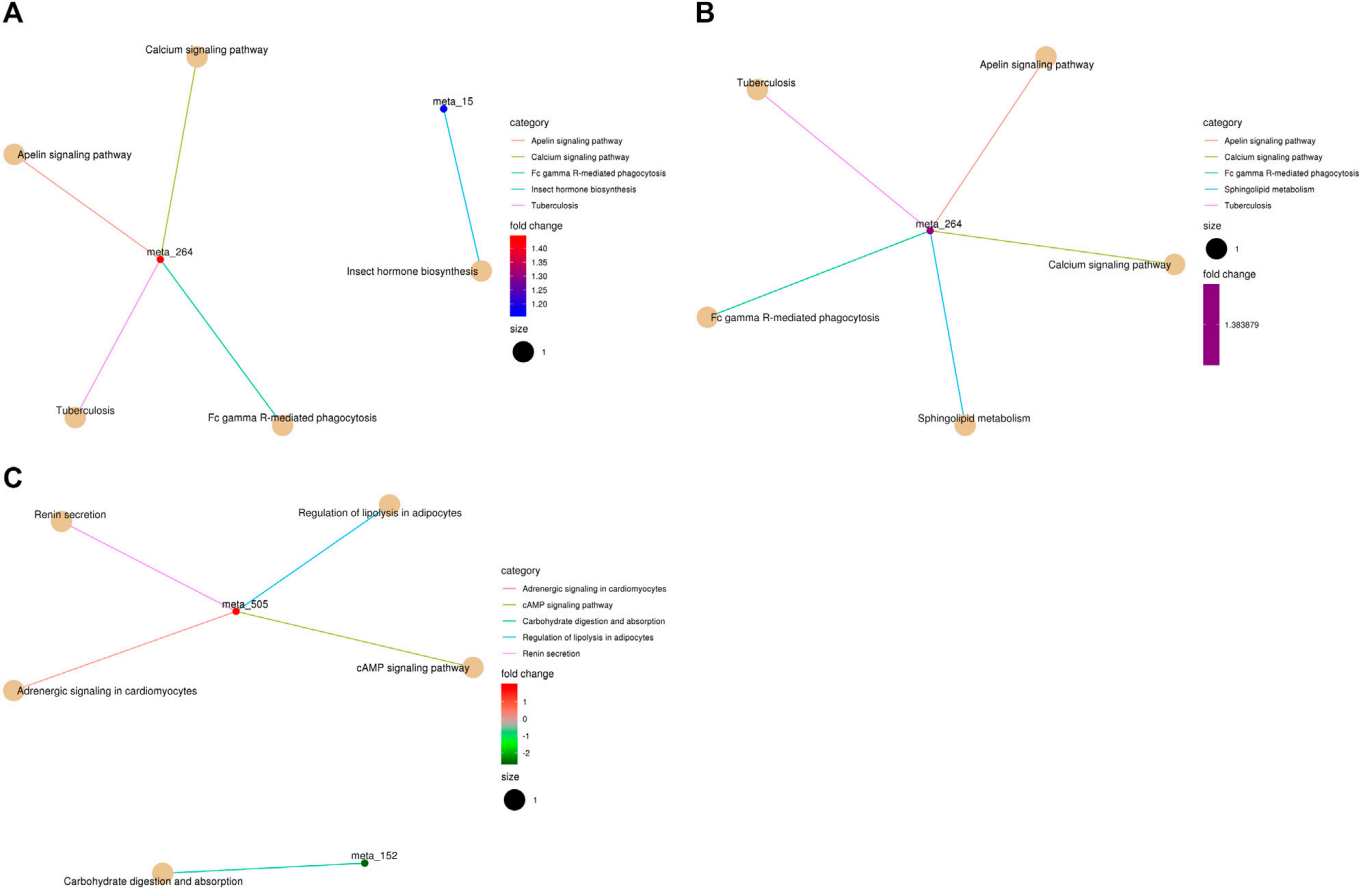
KEGG pathway-enriched cnetplots of differentially abundant metabolites. **(A)**, NT *vs* CS2; **(B)**, NT *vs* CS6; and **(C)**, NT *vs* CS12. (meta_15: farnesy1 pyrophosphate, meta_264: sphingosine-1-phosphate, and meta_505: epinephrine,).

The metabolic pathways of differentially abundant metabolites were explored using the KEGG database. The pathway enrichment analysis of metabolites was performed *via* the R package clusterProfiler. The different metabolic pathways between the NT and CS groups are presented in Figure 4, and network maps of metabolites and metabolic pathways between the NT and CS groups are shown in Figure 5.

Metabolic pathway analysis is very important for metabonomics and useful for identifying the metabolic signals and pathways of metabolites and exploring the related metabolites and genes (Li et al., 2019). There were eight main differentially abundant metabolites involved in three KEGG pathway classifications (metabolism, organismal systems, and environmental information processing) between the NT group and the cold exposure groups. Compared with the NT group, the differentially abundant metabolites of the CS2 group participated in 20 metabolic or biosynthetic pathways in the body; those of the CS6 group participated in 17 metabolic or biosynthetic pathways, and those of the CS12 group participated in 11 metabolic or biosynthetic pathways.

Metabolic pathways and enrichment analyses revealed that most of the altered pathways were related to body metabolism, such as 2-oxocarboxylic acid metabolism, alanine, aspartate and glutamate metabolism, biosynthesis of amino acids, citrate cycle (TCA cycle), glycerophospholipid metabolism, glyoxylate and dicarboxylate metabolism, phenylalanine metabolism, purine metabolism, steroid biosynthesis, terpenoid backbone biosynthesis, and tyrosine metabolism. Cold stress affects the body’s ability to adjust its metabolic functions to adapt to abnormal environmental conditions. Changes in body metabolism were also observed in chickens, pigs, fish, and other animals and plants under cold stress conditions (Lu, et al., 2018; Pan et al., 2018; Nie, et al., 2018; Song et al., 2019; Wang, et al., 2018; Zhou, et al., 2018).

In the present study, compared with the NT group, the most affected metabolic pathway among the three cold exposure groups was associated with amino acid metabolism, which indicated greater body amino acid mobilization and requirements. A study on tilapia also showed that cold stress significantly changed the amino acid levels and classes connected with the metabolism (Li et al., 2020). Zhou et al. (2011) reported that the concentration of five free amino acids in the hepatopancreas increased directly with decreasing temperature. Amino acid metabolism is closely related to a variety of proteins in the body, and it is conducive to maintaining the protein integrity and function and enhancing the adaptation of the body to cold environments. An early study on protein–concentration and environment–temperature interactions reported that pigs showed an increased feed intake at environmental temperatures below 16.5°C and a slight reduction in weight gain (Seymour et al., 1964). Henry (1985) reported that exposure to a cold environment increases the energy requirement of pigs for thermoregulation by stimulating the food intake, which also increases the daily amino acid and protein intake and improves the body amino acid and protein balance during cold stress.

In the current study, the 2-h and 6-h cold exposure groups shared 11 similar pathways (except two pathways), which revealed that these pathways are strongly correlated with short-term cold stress and that the pigs under 2-h and 6-h cold conditions had similar metabolic processes. Most of the pathways were connected with isocitrate (2-h exposure; downregulation) and citric acid (6-h exposure; upregulation), which are important for body energy metabolism. The downregulation of isocitrate and the altered pathways probably contributed to energy consumption under sudden and 2-h cold stimulation. With the extension of cold adaptation and the upregulation of citric acid, the body adapts to meet the needs of energy metabolism to some extent. The altered metabolic pathways of the 12-h cold exposure group were almost different from those of the 2-h and 6-h groups, and most of the pathways were related to epinephrine (tyrosine metabolism, adrenergic signaling in cardiomyocytes, regulation of lipolysis in adipocytes, renin secretion, cAMP signaling pathway, and neuroactive ligand–receptor interaction). Epinephrine is a hormone secreted mainly by the adrenal medulla. Its main function is to increase the cardiac output and blood glucose levels. Studies have shown that cold stress promotes its secretion to adjust the body metabolism (Nachankar et al., 2005). Epinephrine is also related to increased heat production. The upregulation of epinephrine during 12 h of cold exposure altered the relevant pathway and contributed to the adjustment of energy metabolism. Moreover, high-quality and adequate diets are beneficial for improving body energy and protein metabolism and minimizing the risk of diseases under cold exposure, especially for long-term cold exposure (da Silva et al., 2019).

Cold stress can change the body metabolism and impair the immune system (LaVoy et al., 2011). Under cold conditions, more effective heat conservation mechanisms can be developed in response to cold stimuli by adaptive training compared to those under acute or initial cold exposure (Castellani and Young, 2016).

## Conclusion

The results revealed that the metabolite profiles of piglet livers differed under different cold exposure times, and the metabolic pathways were also different. Eight known differentially abundant metabolites were observed between the CS groups and the control group. The KEGG analysis showed that the metabolic pathways of related metabolites were more related to carbohydrates under 2-h and 6-h cold exposure and more related to epinephrine under 12 h of cold exposure.

## Data Availability

The original contributions presented in the study are included in the article/Supplementary Material; further inquiries can be directed to the corresponding author.
